# COVID-19 and young people with intellectual disability: a service review

**DOI:** 10.1192/bjo.2021.877

**Published:** 2021-06-18

**Authors:** Omer Minhas, Pippa Mundy, Jessica Stewart

**Affiliations:** 1 Cwm Taf Morgannwg University Health Board; 2 Cardiff and Vale University Health Board

## Abstract

**Aims:**

A service review of specialist child and adolescent intellectual disability provision in South Wales was conducted between March and May 2020. The purpose was to explore the impact of the first COVID-19 pandemic lockdown on children with intellectual disability and their families. The review aimed to explore if the disruption to the systems involved in their care would impact their wellbeing and behavioural presentations. We also measured if there had been an increase in the use of medication. Our focus was on the distress calls, which are requests for urgent clinical review to prevent a crisis.A service review of specialist child and adolescent intellectual disability provision in South Wales was conducted between March and May 2020. The purpose was to explore the impact of the first COVID-19 pandemic lockdown on children with intellectual disability and their families. The review aimed to explore if the disruption to the systems involved in their care would impact their wellbeing and behavioural presentations. We also measured if there had been an increase in the use of medication. Our focus was on the distress calls, which are requests for urgent clinical review to prevent a crisis.

**Method:**

Six clinical areas across three Welsh health boards under the same specialist team were surveyed. Case notes and email correspondence were reviewed to obtain the number and content of crisis calls made to specialist CAMHS across an eight week period during the first UK COVID-19 lockdown. Data were gathered on frequency, purpose, and outcome of calls. Comparison data were also collected for the period October 2019 to March 2020.

**Result:**

Pre-COVID-19: Two crisis calls were identified in two different areas during the pre-COVID period surveyed. Increases in medication and increases in respite care packages were made as a result.

During COVID-19 restrictions: 20 different initial distress calls made (children age 9 and 17 years old (M = 13.2); 75% were boys) across five of the six clinical areas. Of these 20 calls, 17 were active cases and 3 were new referrals. 95% of calls resulted in medication increases and there were few other interventions used due to COVID-19 constraints. Changes to the child's support system were discussed across all cases and return to school was highlighted as a key protective factor in improved well-being. Differences between clinical areas were also identified.

**Conclusion:**

There was a clear increase in distress calls and requests to prescribe or increase psychotropic medication to calm the distress during the ‘lockdown’. Changes in behavioural presentation may have occurred partly due to the disruption to the complex systems that typically support a child and the shift away from community support. Children with intellectual disability and their families are unique and embedded in complex systems comprising schools, respite, and healthcare provision which work together to deliver optimal mental healthcare with psychosocial interventions with medication for higher-risk situations. Any shifts in these systems may lead to an imbalance and a higher likelihood of medication use.

